# Using latent variables to improve the management of depression among hemodialysis patients

**DOI:** 10.1080/0886022X.2024.2350767

**Published:** 2024-08-01

**Authors:** Douglas D. Gunzler, Jacqueline Dolata, Maria Figueroa, Kelley Kauffman, Julie Pencak, Martha Sajatovic, Ashwini R. Sehgal

**Affiliations:** a Center for Health Care Research and Policy, Population Health and Equity Research Institute, The MetroHealth System, Cleveland, OH, USA; b School of Medicine, Case Western Reserve University, Cleveland, OH, USA; c Center for Health Equity, Engagement, Education and Research, Population Health and Equity Research Institute, The MetroHealth System, Cleveland, OH, USA; d Neurological Institute, University Hospitals Cleveland Medical Center, Cleveland, OH, USA; e Institute for Health Opportunity, Partnership, and Empowerment, The MetroHealth System, Cleveland, OH, USA

**Keywords:** Hemodialysis, depression screening, structural equation modeling, multiple indicator multiple cause modeling, differential item functioning

## Abstract

**Background:**

Screening for depression can be challenging among hemodialysis patients due to the overlap of depressive symptoms with dialysis or kidney disease related symptoms. The aim of this study was to understand these overlapping symptoms and develop a depression screening tool for better clinical assessment of depressive symptoms in dialysis patients.

**Methods:**

We surveyed 1,085 dialysis patients between March 1, 2018 and February 28, 2023 at 15 dialysis facilities in Northeast Ohio with the 9-item patient health questionnaire (PHQ-9) and kidney disease quality of life (KDQOL) instrument. To evaluate overlap across questionnaire items, we used structural equation modeling (SEM). We predicted and transformed factor scores to create a hemodialysis-adjusted PHQ-9 (hdPHQ-9). In exploratory analysis (*N* = 173), we evaluated the performance of the hdPHQ-9 relative to the PHQ-9 that also received a Mini-International Neuropsychiatric Interview.

**Results:**

Our study sample included a high percentage of Black patients (74.6%) and 157 (14.5%) survey participants screened positive for depression (PHQ-9 ≥ 10). The magnitude of overlap was small for (respectively, PHQ-9 item with KDQOL^TM^ item) fatigue with washed out, guilt with burden on family, appetite with nausea and movement with lightheaded. The hdPHQ-9 showed reasonably high sensitivity (0.81 with 95% confidence interval [CI] 0.58, 0.95) and specificity (0.84 with 95% CI 0.77, 0.89); however, this was not a significant improvement from the PHQ-9.

**Conclusion:**

There is little overlap between depressive symptoms and dialysis or kidney disease symptoms. The PHQ-9 was found to be an appropriate depression screening instrument for dialysis patients.

## Introduction

Major depressive disorder (MDD) is the most common mental health disorder among hemodialysis patients [1]. The prevalence of MDD among dialysis patients has been estimated at approximately 20–30% [2–5]. MDD is associated with poor health outcomes, including poor adherence to treatment, inadequate nutrition, increased hospitalization, and premature death [2,6–9]. Comorbid depression has a negative impact on quality of life in chronic kidney disease (CKD) patients [10].

Dialysis patients commonly experience depressive symptoms. However, clinicians cannot reliably determine if symptoms such as fatigue, appetite problems, cognitive impairment, and functional impairment are due to depression or are a result of dialysis treatment or underlying kidney disease (hereafter referred to as dialysis-related symptoms). This can lead to inappropriate clinical decisions (e.g., medication selection or escalation) and to false-positive or false-negative inferences regarding treatment effectiveness. Some patients who “should” respond to certain anti-depressants may not because their presenting symptoms are dialysis-related symptoms rather than mental health symptoms. In seeking deeper knowledge of symptom origins, we apply methods which should improve our ability to assess depression and changes in the depression syndrome in dialysis patients. Our research hypothesis is that if we adjust the scoring of a depression screener after accounting for the overlap of depressive symptoms with dialysis-related symptoms in a study population of dialysis patients, that the adjusted screener will more accurately screen for depression as compared to a standard screener.

A prior study by our team used similar methods to evaluate overlapping symptoms of multiple sclerosis (MS) and depression and subsequently develop an adjusted depression screening tool [11–13]. We evaluated 3,507 MS patients with a self-reported depression screening (PHQ-9) score and found significant overlap in fatigue, cognitive impairment and functional impairment. However, that study was completed with observational data and there was no available clinical assessment to study the performance of our screening instrument. This study applies this methodology in a sample of dialysis patients collected prospectively and then conducts exploratory data analysis to address the performance of the instrument. While there was much prior literature concerned with measurement bias in depression screening in MS patients [14–19] to our knowledge, there is limited prior studies concerned with measurement bias in depression screening in dialysis patients. For example, AlAwwa et al. were concerned with the overlap between the somatic symptoms of depression and those of uremia seen in end-stage renal disease patients, which could affect depression diagnosis [20]. In a small study of 163 participants the team found only modest differences in depression scores among dialysis patients before and after dialysis, with a trivial effect of uremic symptoms on the diagnosis of depression[20].

The PHQ-9 is a short, practical and self-rated screen for depression in both community and clinical samples [21]. In this study, we focus on better understanding how the PHQ-9 might be applied in dialysis patients. While a study by Watnick et al. validated the use of the PHQ-9 in dialysis patients [3], our study builds upon such prior work by 1) using a much larger sample of dialysis patients and 2) conducting psychometric analysis of the effect of dialysis-related symptoms on the measurement properties of the PHQ-9. In exploratory data analysis, we test the performance of our adjusted instrument relative to the PHQ-9 in a subsample of our study participants who were given Mini-International Neuropsychiatric Interview (MINI), a structured diagnostic instrument that is a gold standard for clinical diagnosis of depression in research settings [22].

## Methods

### Setting and participants

The study was conducted at 17 in-center hemodialysis facilities that are part of the Centers for Dialysis Care (CDC) system in Northeast Ohio. Patients were identified from these facilities using our inclusion/exclusion criteria described below from the CDC Clarity database. All adult, English-speaking, cognitively intact patients who had been on maintenance hemodialysis for at least 3 months were invited to participate in the study. The median facility size is 92, 47% of patients are female, 48% are African-American, and 3% are Hispanic. These facility and patient characteristics are roughly comparable to national figures, except that there are more African American and fewer Hispanic dialysis patients in the Cleveland area compared to the United States as a whole. Any patient not at a CDC unit was excluded from the study. Children age < 18 were excluded from the study because the performance of the PHQ-9 and the DSM-5 criteria for depression are slightly different among children compared to adults [23,24]. We excluded new patients because the first 3 months of dialysis treatment is often a time of multiple adjustments in dialysis prescriptions which may in turn affect dialysis complications. Non-English speaking patients were excluded from the study because we were not budgeted to have study documents in both English and Spanish. Finally, cognitively impaired patients were excluded from the study because they will be unable to reliably report on depressive symptoms and dialysis complications [25].

We did not remove patients on anti-depressants. We needed to observe the full range of depression levels for our algorithm and depressed patients are more likely to already be treated for depression (unless it’s newly diagnosed). In addition, standard practice is to administer PHQ-9 to all patients, regardless of whether they are on anti-depressants or not. Similarly, we wanted to examine all patients in our study.

The total number of patients screened by chart was *N* = 2357. Out of that total, *N* = 1272 were excluded (27 non-English speaking, 43 death, 756 refused and 446 our team was unable to contact). We collected data on 1085 study participants between March 1, 2018 and February 28, 2023.

Pre-COVID-19 pandemic (March 1, 2020) the quality of life and depression screening surveys under study (see *Measures Assessed*) were applied and taken in person during the dialysis sessions. The surveys were verbally administered by a study coordinator. Post-COVID-19 pandemic the study procedures changed. Letters were sent out to all eligible patients at the 17 in-center hemodialysis facilities introducing the study. Surveys were then administered *via* a phone call by a study coordinator during or after dialysis sessions.

All study participants enrolling in the study after March 1, 2020 (a subsample of 173) were evaluated by the study psychiatric nurse practitioner (KMK) using the Mini International Neuropsychiatric Interview. Our study team considered using this sample as strictly a testing sample, with the remaining 912 participants to be used for model building. However, the timing of the collection of this subsample ended up occurring during the COVID pandemic. We found significantly higher scores in anxiety (using the General Anxiety Disorder-7 [2]) and depression screening in this subsample compared to depression screening in participants pre-COVID (see Supplementary Table). As a result, rather than using these data as a testing sample only, we combined data for the model building such that the full range of scores pre- and post-COVID could be observed in practice.

Before March 1, 2020, some other patients with a moderate to high PHQ-9 were also given the Mini International Neuropsychiatric Interview for potential enrollment in a trial of weekly, directly observed fluoxetine treatment [1]. Patients with MDD based on the Mini International Neuropsychiatric Interview and meeting inclusion criteria as discussed by Kauffman et al. [1] were invited to participate in the trial. Despite having an initial patient recruitment goal of 96 participants, only 16 were enrolled in this feasibility study. This resulted primarily from more patients than expected scoring below our thresholds on the PHQ-9 (even after expanding our threshold for depression screening, Kauffman et al. [1]) and/or being on a psychiatric medication before enrollment. All participants provided written informed consent, and the study was approved by the institutional review board of the MetroHealth System, Cleveland, Ohio (IRB17-00768).

### Measures assessed

The PHQ-9 is a brief, self-report depression severity measure [21]. Patients specify frequency in the past 2 weeks (0 = not at all to 3 = every day) of nine symptoms, yielding a total score (range: 0–27). Scores on this self-reported instrument are often used to guide treatment decisions [21]. In particular, a PHQ-9 ≥ 10 has been previously established as a screening cutoff for depressive disorder. The Mini-International Neuropsychiatric Interview (MINI), has been established and accepted in psychiatric care as a structured interview for diagnosis of depression [22].

The Kidney Disease Quality of Life^TM^ (KDQOL^TM^) survey is a kidney disease-specific measure of health-related quality of life (HRQOL) [26]. The long form of the KDQOL (134 items) was developed in 1994 by the Kidney Disease Quality of Life Working Group with support from Amgen. In our study we used specific items from the long form of the KDQOL: item 15 (frustrated), item 16 (burden to family), item 19 (nocturnal cramps), item 23 (lightheaded), item 26 (washed out), item 27 (nausea), and item 37 (leg swelling). Higher scores on each item listed above indicated worse impairment, except for Items 15 and 16 and thus we reverse coded those two items. In the appendices we include sample questionnaires of both the PHQ-9 and the Quality of Life Questionnaire our study coordinators administered to participants. The Quality of Life Questionnaire includes 49 items from the KDQOL as well as the General Anxiety Disorder-7 (listed here as items 42–48). Demographic variables incorporated in our analysis plan included age, race (white, black, other) and gender.

### Overview of the statistical approaches

Structural equation modeling (SEM) enables researchers to examine the overlap of depressive vs. dialysis-related symptoms [11–13,27,28]. SEM is a flexible multivariate technique that allows relationships among latent variables and observed variables to be examined. A latent variable is a variable that is assumed to be not directly observed and instead is inferred using observed variables. For example, as a depression screening tool, the nine items of the PHQ-9 can be used to infer a latent variable of *depression*, since these items are assumed to describe different symptoms of depression and in aggregate depression. Note that we italicize *depression* here (and throughout this paper) to denote we are referring to a latent variable rather than the condition in itself. In particular a latent variable model, also termed a confirmatory factor analysis (CFA) model, relates a latent variable to the observed variables postulated to infer the latent variable. In such a model random measurement error represents the difference between what is assumed to be the true score, the latent variable, and the observed variables. One does not have to assume a single latent variable, as sometimes multiple latent variables are inferred for a given set of observed variables.

An extension of the basic CFA model as described above is the multiple indicator multiple cause (MIMIC) model. The MIMIC model allows one to incorporate covariates in the latent variable model and regress the latent variable on the covariates. The MIMIC model also can permit detection and adjustment for differential item functioning (DIF) by regressing the covariates on the observed variables used to infer the latent variable. DIF occurs when people from different groups with the same score on a latent variable have a different probability of giving a certain response on a questionnaire or test. For example, in our study, consider two participants one experiencing dialysis-related nocturnal cramps and one not experiencing such cramps. Both these participants can still have the same overall level of depression. Yet, these two participants have a different probability of experiencing a sleep disturbance, since experiencing nocturnal cramps are postulated to lead to greater sleep disturbance. In short, using the MIMIC model with DIF in our study we are able to update our original estimate of self-report depression *via* the PHQ-9 taking into account overlapping symptoms of dialysis and depression.

### Statistical analyses

We reported descriptive statistics as mean (standard deviation) for continuous measures and number of subjects (percentage) for each category for discrete measures to summarize demographic information and scores on our measures of depression screening, depression diagnosis and the KDQOL items. We reported from ANOVA test and chi-square test where appropriate for assessing statistical significance in the comparison of the distributions of the data across PHQ-9 screening strata.

Our goal in our statistical analyses is to develop an adjusted PHQ-9 score, which we label as the hdPHQ-9, for each participant that takes into account the overlap of dialysis-related symptoms and depressive symptoms. Since the hdPHQ-9 is a re-assigned PHQ-9 for each participant determined by the amount of overlap as calculated in a statistical model using currently available PHQ-9 and KDQOL data, no new questions are involved. In exploratory analysis (*N* = 173), we then compare the performance of the hdPHQ-9 relative to the PHQ-9 that also received a MINI clinical assessment of depression.

**Figure 1.**
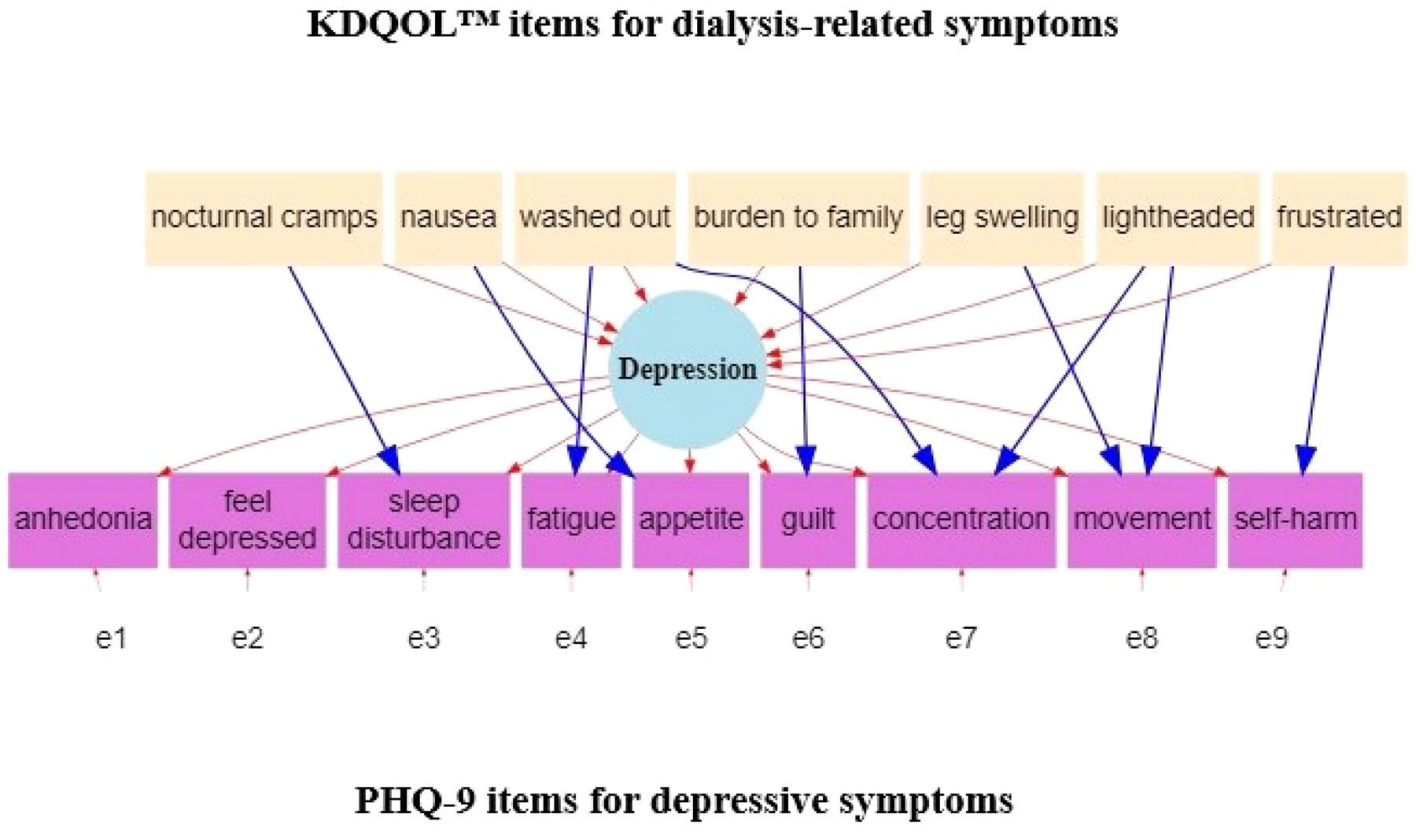
Model for analysis of overlapping depressive symptoms and dialysis-related symptoms. There are pairwise correlations among all the KDQOL items (nocturnal cramps, nausea, washed out, burden to family, leg swelling, lightheaded and frustrated) in the specified model that we omit from the figure due to visual complexity.

To clarify, the hdPHQ-9 is our term for an individual level score, a hemodialysis-adjusted PHQ-9, calculated using our statistical model and algorithm. The conceptual model of the idea under study and corresponding diagram (see Figure 1) depicts the overlap across specific items of the PHQ-9 and KDQOL. This conceptual model is then translated into a formal statistical model. The PHQ-9 and KDQOL are used as data for the statistical model and algorithm. The output score, hdPHQ-9, can be interpreted as an algorithmic-adjusted PHQ-9 score with the same empirical distribution as the PHQ-9 within our study sample. Thus, for practical purposes, a clinician would not be giving any new tests, but rather the PHQ-9 and KDQOL as directed. It is our statistical approach and algorithm that is using these questionnaires to calculate a hdPHQ-9.

We outline our approach to determining a hdPHQ-9 score for each participant below. For starters, using a similar approach as described by Gunzler and Morris [12] we worked with a team of specialists (nephrologists, psychiatrists, nurses and other methodologists) to develop a conceptual model and subsequent path diagram (see Figure 1) that describes the overlap of depressive symptoms and dialysis-related symptoms.

The MIMIC model represented in Figure 1 assumes that PHQ-9 items for sleep disturbance, fatigue, appetite, guilt, concentration, movement (i.e., for clarification, movement being slowed down or sped up by a mental health condition) and self-harm have the potential to overlap with dialysis-related symptoms as described by items from the KDQOL. Namely, within Figure 1, we assume a unidimensional latent construct of *depression*. Measurement error (symbolized as e1 through e9) are the differences between the items of the PHQ-9 and *depression*. Dialysis-related symptoms then are hypothesized to influence *depression* overall as well as specific PHQ-9 items in the form of DIF (i.e., overlap). For example, we hypothesize, a higher level of nausea due to dialysis will lead to higher depression overall, but also will lead directly to more appetite problems which underly the level of depression. The coefficients of overlap (i.e., DIF paths) that are reported are based on a standardized scale and generally the effect size can be interpreted in the range of null (0.0), small (0.2), medium (0.5), large (0.8) and the largest possible (1.0) effects [29].

In order to test the overall plausibility of our hypothesized model in Figure 1 for the data, we evaluated the model fit using the chi-square, comparative fit index (CFI), Tucker Lewis fit index (TLI), root mean square error of approximation (RMSEA) and standardized root mean square residual (SRMR) statistical test values [30–33]. A nonsignificant chi-square value, CFI and TLI ≥ 0.95, RMSEA value of ≤ 0.05 and SRMR value ≤ 0.05 represent an excellent fitting model, while a smaller chi square test statistic, CFI and TLI ≥ 0.90, RMSEA value of ≤ 0.08 and SRMR value ≤ 0.08 represent an acceptable fitting model [13]. RMSEA, CFI, TLI and SRMR values should all reach acceptable (preferably excellent) levels before designating a model as good fitting [13]. The RMSEA statistic is especially useful in that it provides a confidence interval for our point estimate.

We also extended the MIMIC model to include the regression of *depression* on additional covariates of age, gender (male, female) and race (Black, all other races) to evaluate for differences in the level of depression across these groups. If found to be statistically significant, we would incorporate any group differences in our final model.

Factor scores are individualized scores on *depression* that can be predicted from the model using a Bayesian approach [13]. Accounting for all relationships in Figure 1 adjusts these factor scores in *depression* in a similar manner to how multiple regression can adjust the estimated effect of a treatment when controlling for confounders. The distribution of the factor scores depends on the ability of the items to measure the full range of the latent variable, the items’ measurement parameters, and the mean and variance specified in the model. Therefore, our team had previously developed methods, an application of a probability integral transform [34], to transform factors scores back to the original scale of the PHQ-9 [11–13]. The assumption of this method was that given a large enough sample, the empirical distribution of scores were the same for both the PHQ-9 and hdPHQ-9. Importantly, however, particular individuals in our study were re-assigned a new score on the hdPHQ-9 that was higher or lower than their previous score on the PHQ-9.

In exploratory data analysis we checked model performance. In the subsample (*N* = 173) with both a PHQ-9 and MINI clinical assessment, we compared the performance of the hdPHQ-9 to the PHQ-9 using the screening cutoff for major depression of 10 on both scales. Here we set up a 2 by 2 contingency table based on positive and negative diagnosis and screening for each scale and calculated the sensitivity, specificity, positive predictive value and negative predictive value with 95% confidence intervals. We also use receiver operating characteristic (ROC) curve analyses based on maximizing the area under the curve (AUC) to determine what is the optimal cutoff for major depression in this study sample for both the PHQ-9 and hdPHQ-9.

The PHQ-9 items have four levels and right skewed, therefore treated as an ordered-categorical variable. We used a mean and variance adjusted weighted least squares estimator (WLSMV option in MPlus) in our models to assess model fit. However, in order to generate factor score, we use the same final model with a maximum likelihood estimator with robust standard errors (MLR option in MPlus) in our analyses (Múthen and Múthen, 2013). MLR option allowed us to generate a factor score in all cases, while the WLMSV option omitted 14 cases due to a small amount of missing data. This step was acceptable given our model using the MLR option had similar results as our model using the WLSMV option. Also, due to sample size and since each of the PHQ-9 items has at least 4 ordinal categories, the measures can be viewed as approximating an interval scale [35–38].

We define α = 0.05 for our level of significance in all statistical tests. All statistical tests are two-tailed. SEM was carried out using MPlus version 8.6. R program in the R studio environment was used for data management, graphical displays and other descriptive analyses and the MPlusAutomation package helped automate SEM estimation and interpretation [39].

## Results

### Descriptive analyses

The study sample was 47.6% female, 74.6% Black, and had an average age of 65.6 years (SD = 13.7) as shown in Table 1. Similar to national reports, men outnumber women and the study sample is on average of an older age [40,41]. The percentage of Black patients available in this study sample (of our overall sample 38.2% Black Male, 36.3% Black Female) was much higher than other large studies, e.g., Myers et al. [40] and Kucirka et al. [42]). More generally, about one in three people receiving dialysis are Black [43]. To help understand race by gender differences across level of PHQ-9 in our study sample, we ran a linear regression analysis of the PHQ-9 total score on race, gender and the interaction of race by gender (i.e., Black Female, Black Male, Other Female, Other Male, White Female, White Male). No effects were found to be significant.

**Table 1.** Baseline characteristics of dialysis study population.

			PHQ-9 ≥ 10						
	Overall		No		Yes		*p*	MINI Subsample	
n	1085		928		157			173	
Female (%)	516	(47.60)	435	(46.90)	81	(51.60)	0.313	77	(44.50)
Race (%)							0.182		
Black	809	(74.60)	701	(75.50)	108	(68.80)		113	(65.30)
Other	8	(0.70)	7	(0.80)	1	(0.60)		4	(2.30)
White	268	(24.70)	220	(23.70)	48	(30.60)		56	(32.40)
Age	65.55	(13.67)	66.1	(13.75)	62.27	(12.74)	0.001	62.52	(12.83)
PHQ-9 Total Score	4.55	(4.66)	2.99	(2.48)	13.76	(3.82)	<0.001	6.18	(5.07)
MINI Depression (%)								20	(12.10)
KDQOL ^TM^									
Frustrated (reverse coded)	3.26	(1.66)	3.49	(1.59)	1.89	(1.32)	<0.001	2.66	(1.65)
Burden to family (reverse coded	3.86	(1.56)	4.09	(1.42)	2.53	(1.68)	<0.001	3.33	(1.68)
Nocturnal cramps	2.04	(1.19)	1.95	(1.12)	2.58	(1.41)	<0.001	2.37	(1.34)
Lightheaded	1.13	(0.56)	1.11	(0.52)	1.24	(0.76)	0.005	1.18	(0.65)
Washed out	2.12	(1.22)	1.89	(1.05)	3.46	(1.33)	<0.001	2.55	(1.34)
Nausea	1.61	(1.02)	1.49	(0.87)	2.29	(1.46)	<0.001	1.86	(1.23)
Leg swelling	1.7	(1.09)	1.54	(0.93)	2.61	(1.47)	<0.001	1.84	(1.24)

mean ± standard deviation for continuous measures and number of subjects in each category for discrete measures with *p*-values reported from ANOVA and chi-square tests where appropriate.

Nearly 14.5% (*n* = 157) of patients had PHQ-9 ≥ 10 at their entry. The distribution of PHQ-9 scores represents a wide range of depression screening severity levels (Supplementary Figure S1). However, aforementioned, the prevalence of MDD among dialysis patients has been estimated at approximately 20–30% [2–5]. The percentage of patients screening positive for depression *via* the PHQ-9 in hemodialysis patients has been found to be similarly high. For example, Agrawaal et al. (2019) found the prevalence to be 37% in a study sample of 100 with stage 5 CKD at a tertiary care center and Anderson et al. found the prevalence to be 26.4% in a study sample of 485 [44].

We summarize the characteristics of the study sample by the PHQ-9 binary threshold of 10 (screening cutoff for depressive disorder) in Table 1, with significant differences across age and the KDQOL ^TM^ item scores. We also describe the subsample of 173 received both depression screening (Yes = 23.1%) and Mini-International Neuropsychiatric Interview for clinical assessment for depression (Yes = 12.1%) in Table 1. Positive depression screening is higher in this subsample compared to the overall study sample. Other studies have also found that positive depression screening *via* the PHQ-9 increased during the COVID pandemic, for example Ettman et al. [45].

### MIMIC model to examine and correct for the overlap of depressive symptoms with dialysis-related symptoms

We analyzed the MIMIC model represented in Figure 1. Model fit was acceptable to excellent (see Table 2 Legend). Significant overlap was found for the PHQ-9 items for fatigue, appetite, guilt, and movement with KDQOL^TM^ items (see Table 2). For model comparison, if we constrain all DIF paths in the MIMIC model to zero (i.e., an assumption of no overlap), the chi-square difference test is significant (*p* < 0.001) and the model fit is worse in all criterion (see Table 2 Legend). The magnitude of estimates of overlap are small for fatigue with washed out, guilt with burden on family, appetite with nausea and movement with lightheaded and not significant otherwise.

**Table 2.** Standardized MIMIC model estimates.

		DIF (overlap) Paths			DIF PATHS CONSTRAINED TO ZERO		
		Estimate	SE	*p*	Estimate	SE	*p*
Depression	BY						
	Anhedonia	0.75	0.02	<0.001	0.75	0.02	<0.001
	Feel depressed	0.86	0.02	<0.001	0.86	0.02	<0.001
	Sleep disturbance	0.64	0.03	<0.001	0.65	0.03	<0.001
	Fatigue	0.58	0.03	<0.001	0.70	0.03	<0.001
	Appetite	0.58	0.03	<0.001	0.61	0.03	<0.001
	Guilt	0.69	0.03	<0.001	0.78	0.03	<0.001
	Concentration	0.72	0.04	<0.001	0.71	0.03	<0.001
	Movement	0.60	0.05	<0.001	0.64	0.04	<0.001
	Self-harm	0.74	0.07	<0.001	0.75	0.05	<0.001
Depression	On						
	Burden to family	0.20	0.03	<0.001	0.23	0.03	<0.001
	Nocturnal cramps	0.05	0.03	0.078	0.05	0.03	0.067
	Lightheaded	0.01	0.03	0.688	0.03	0.03	0.364
	Washed out	0.25	0.03	<0.001	0.28	0.03	<0.001
	Nausea	0.09	0.03	0.001	0.10	0.03	<0.001
	Leg swelling	0.14	0.03	<0.001	0.14	0.03	<0.001
	Frustrated	0.27	0.03	<0.001	0.25	0.03	<0.001
Sleep disturbance	ON						
	Nocturnal cramps	0.00	0.03	0.914	0		
Fatigue	ON						
	Washed out	0.23	0.03	<0.001	0		
Concentration	ON						
	Washed out	−0.07	0.05	0.126	0		
	Lightheaded	0.03	0.04	0.521	0		
Appetite	ON						
	Nausea	0.10	0.03	0.005	0		
Guilt	ON						
	Burden to family	0.20	0.03	<0.001	0		
Movement	ON						
	Lightheaded	0.09	0.03	0.006	0		
	leg swelling	0.05	0.05	0.246	0		
Self-harm	ON						
	Frustrated	−0.02	0.13	0.882	0		

Model Fit for Model with DIF (Overlap) = Chi-square 156.27 (degrees of freedom = 74) *p* < 0.001; RMSEA 0.032 (90% CI = 0.025, 0.039); CFI = 0.960; TLI = 0.946; SRMR = 0.044.

Model Fit for Model without DIF = Chi-square 244.26 (degrees of freedom = 83) *p* < 0.001; RMSEA 0.043 (90% CI = 0.036, 0.049); CFI = 0.922; TLI = 0.906; SRMR = 0.046.

When we extended the MIMIC model to account for differences in *depression* level by age, race and gender, we found no meaningful difference in model fit and these demographic covariates were not significant. Thus, we did not incorporate these demographic covariates in our final model.

In summary of this section, we find small overlap of depressive symptoms with dialysis-related symptoms that may be inflating or deflating a participants’ PHQ-9 total score. Simultaneously, the MIMIC model is estimating the overlap and then correcting the latent factor of *depression.* For example, we can calculate the small, but significant, effect of this overlap on the factor loadings across the two models in Table 2; the absolute relative percent change in the association between item and the *depression* factor is 17% for fatigue, 13% for guilt, 5% for appetite, and 6% for movement.

### Analysis of hdPHQ-9 compared to PHQ-9

Factor scores in our study represent continuous adjusted depression screening scores for dialysis patients, accounting for the overlap of depressive symptoms with dialysis-related symptoms. Factor scores were then transformed to a corresponding PHQ-9 score, to create the hdPHQ-9 for each participant. In Table 3 we compare the performance of PHQ-9 depression screening with hdPHQ-9 depression screening using a positive screening cutoff of 10 on the MINI subsample. The point estimates are reasonably high (above 0.80) for the sensitivity and specificity for hdPHQ-9. The point estimate for sensitivity is higher for the hdPHQ-9 compared to PHQ-9 and specificity is trivially higher for the hdPHQ-9 compared to PHQ-9. However, this difference in sensitivity is not statistically significant as the point estimates are within the wide 95% confidence intervals of each other.

**Table 3.** Comparison of PHQ-9 and hdPHQ-9 in depression screening against the MINI diagnosis. A. PHQ-9

PHQ-9		MINI Diagnosis	
		+	−
Screening	+	14	26
	−	7	126

Sensitivity: 0.67 ([95% Confidence Interval] 0.43, 0.85); Specificity: 0.83 (0.76, 0.89); Pos Pred Value: 0.35 (0.21, 0.52); Neg Pred Value: 0.95 (0.89, 0.98).

### Optimal screening for depression using the PHQ-9 and hdPHQ-9

Using ROC analyses and the ROC function in Epi in R, we determined the optimal cutoff for depression screening to maximize the area under the curve (AUC) for the PHQ-9 and hdPHQ-9. For the PHQ-9 a score of 8 resulted in a sensitivity = 0.91, specificity = 0.74 and AUC = 0.89, while in the hdPHQ-9 a score of 9 resulted in a sensitivity = 0.91, specificity = 0.78 and AUC = 0.91.

## Discussion

The results of our SEM-based analyses show that there is a small amount of overlap of depressive symptoms with dialysis-related symptoms, namely fatigue, appetite, guilt, and movement. Based on these analyses, the PHQ-9 was found to be an acceptable screen for the management of depression in dialysis patients. No previous study directly evaluated and corrected for the overlap of the PHQ-9 with dialysis in a population of dialysis patients.

For starters, we found the prevalence of depression lower than anticipated (14.5% screened positive overall in the sample of 1085, 12.1% positive diagnosis *via* the MINI in the subsample of 173) in this dialysis study population. For example, the prevalence of MDD in dialysis patients had been estimated at approximately 20–30% [2–5]. and the percentage of patients screening positive for depression *via* the PHQ-9 in hemodialysis patients has been found to be similarly high.

The percentage of Black patients available in this study sample (of our overall sample 38.2% Black Male, 36.3% Black Female) was much higher than other large studies, e.g., Myers et al. [40] and Kucirka et al. [42].

Some past studies have found that African-American men may generally under self-report depression. Black men have been found to have a lower prevalence of MDD than black women and their male counterparts [46–48]. Plowden et al. [49] suggested that depression in African American men may be underreported due to hesitancy in treatment seeking and differential symptom presentation. Further, Perez et al. [50] reported that Black women with depressive symptoms more often report sleep disturbances, self-criticism, and irritability than stereotypical symptoms such as depressed mood. Endorsing somatic symptoms of depression rather than feelings of depression may in itself lead to underscoring of the PHQ-9. Thus potentially these differences in the presentation and reporting of depression screening in Black patients could be impacting a scale like the PHQ-9 in our sample.

In our study sample, many of our patients were on an antidepressant. Some of our participants may be on an antidepressant for reasons other than depression (e.g., sleep, pain, appetite). Both PHQ-9 and KDQOL^TM^ scores can be affected by such treatment.

Further, our clinicians have reported anecdotally a level of acceptance observed in some study participants after adjustment to being on dialysis and associated lifestyle changes that could lead to a decrease in depressive symptoms. We note too that symptoms experienced may not be related to dialysis or depression but due to other comorbidities or life events that we did not model in this study. We were limited to information collected prospectively regarding dialysis treatment and mental health assessment as per our study protocol and did not have more extensive patient history regarding hospitalization, surgery and infections.

There is also the potential that higher inflammatory markers (of which measurement is beyond the scope of this study) explain the associations across depressive symptoms and dialysis-related symptoms. Hyperactivity of the Stress and Hypothalamic-Pituitary-Adrenal (HPA) axis, may exert a possible modulatory influence on MDD [51]. Moreover, there is the possibility that participants with higher inflammatory modulators levels may be at higher risk to develop treatment resistance, thus maintaining a high PHQ-9 score in spite of treatment [52].

Extending our MIMIC model to account for all such sources of confounding would be limited by the data available and unreasonable in practice. In our study, we merely aim to advance the scientific dialogue by accounting for the effect of dialysis-related symptoms in the psychometric analysis of the PHQ-9.

In regard of this more nuanced discussion, we postulate three possibilities regarding the use of the PHQ-9 in our sample. 1) The psychometric properties of the PHQ-9 hold up in lieu of any overlapping dialysis-related symptoms. 2) The PHQ-9 was fine to use in our sample because these particular study participants had a low prevalence of depressive symptoms, which may not be representative of a different sample of hemodialysis patients. Research remains to be done regarding the psychometric analysis of the PHQ-9 in lieu of overlapping dialysis-related symptoms in a study sample with a higher prevalence of depression screening. 3) There is a high proportion of Black patients in this sample, of which symptom presentation may be different and Black patients may underreport the level of depressive symptoms, leading to inherent measurement bias in our use of the PHQ-9. We did not find a significant association between the PHQ-9 and race in supplementary regression analysis or extended MIMIC analysis. However, those findings do not discount that there may be more systematic selection bias in this study sample at a city-wide hospital in which depressive screening could be underreported.

In exploratory data analysis, we found reasonably high sensitivity and specificity for hdPHQ-9, but did not find a significantly improved performance in the hdPHQ-9 compared to the PHQ-9. This result may be a limitation of our subsample size as our 95% confidence intervals were wide. We did find in the point estimate an improved ability for positive screening when an individual had a depression diagnosis (i.e., sensitivity) in the hdPHQ-9, further study is warranted. Despite this negative finding, the calculation of the hdPHQ-9 still proves advantageous in practice. The overall approach leads to a useful tool for helping to determine the amount of measurement bias in the PHQ-9 due to overlapping dialysis-related symptoms for the management of depression. The approach can be replicated for assessment of samples that have more diversity of depressive symptom severity or with other depression screening scales or additional symptoms. Findings might be different in future studies.

The optimal cutoff for positive depression screening for the hdPHQ-9 in this study sample of nine *via* ROC analyses was also found to be slightly closer to the cutoff used in practice of 10 as compared to the PHQ-9 (cutoff of eight found *via* ROC analyses). More generally, future analysis can help evaluate if a lower cutoff would lead to fewer false negative and positive results in depression screening when using the PHQ-9 in a dialysis patient population.

The assumptions of transforming the algorithm and matching with the empirical distribution of PHQ-9 scores is that within this sample of dialysis patients, we have correctly identified the empirical distribution (mean, standard deviation, range and cutoffs) of the PHQ-9, but have misclassified the particular scores of individual patients. This work further assumes that we correctly specified our MIMIC model and that some symptoms are characteristic of only depressive symptoms (such as anhedonia and feel depressed) while some depressive symptoms overlap with dialysis-related symptoms. We tested items of the PHQ-9 that theoretically had an overt possibility of overlap with dialysis-related symptoms within our data base. As a result, we assumed that high endorsement of such overlapping items across questionnaires inflates depression screening scores.

Aforementioned, an important study limitation that may have impacted our findings is that we found a lower than expected positive depression screening in our study sample. Given a higher prevalence of depression, we may have found a larger amount of overlap and our algorithm for the hdPHQ-9 may have found more discrepancy in screening compared to the PHQ-9, such as found in our previous study with an MS study sample and depression screening prevalence of approximately 30% [11–13]. Our team also did not do the MINI depression diagnosis on all study participants; thus, our performance evaluation of hdPHQ-9 relative to the PHQ-9 was on a subsample of 173 and was considered merely exploratory. This subsample collection also occurred post-COVID in which we observed a higher percentage of positive depression screening cases as compared to our full sample. Thus, another limitation is that this subsample is a nonrandom representation of our study population.

A limitation to using the MIMIC model is that they test for uniform, but not nonuniform DIF [53]. Uniform DIF is constant across *depression*, while non-uniform DIF varies across *depression*. We addressed this limitation through extended MIMIC analyses for group differences by race, gender and age. We did not find any significant group differences in this study sample.

We summarize again here for reader clarity three major potential limitations of our work. A potential limitation of our analyses is selection bias in our sample leading to a low prevalence of depression screening. Another limitation is that this subsample is a nonrandom representation of our study population. Finally, a third major limitation is that the statistical model is assumed to be correctly specified (we correctly modeled all sources of overlap in the PHQ-9 with dialysis-related symptoms) which in practice is not possible and limited by available data.

Other measures, for example anxiety, potentially overlap with depression screening and are important to examine in hemodialysis patients, requiring future extension of our models. These approaches show promise for clinicians for improved use of patient-reported outcomes in patients with comorbid conditions, regarding better diagnosis, treatment decisions and inferences about effectiveness of treatment.

As a result of this study, in practice, clinicians should proceed with some caution when interpreting a test, questionnaire or other scale of interest in lieu of other comorbidities in patient care management. Our models evaluated comorbid dialysis-related symptoms and depressive symptoms and we found small overlap that did not significantly impact our depression screening *via* the PHQ-9. However, a dialysis patient could present with several comorbidities (e.g., anxiety, depression and diabetes) that could interact in a complex manner and symptom presentation could be typical or atypical. As opposed to just viewing depression screening as a binary yes or no decision, clinicians should consider that continuous scores close to a screening cutoff (either above or below) may be inflated or deflated due to presence or absence of overlapping symptoms. Particular attention should be given to what symptoms a patient is endorsing (e.g., fatigue or appetite problems) and considering whether such symptoms could also be due to other comorbidities. For example, in determining endorsement of movement problems as a depressive symptoms, there should be clarity that these problems stem from mental health and not physical activity as we would more typically understand it. We recommend considering both empirical scoring and clinical judgment of comorbidities and symptomatology when interpreting patient-reported outcomes and making overall care decisions.

## Conclusion

Our objective was to determine if the PHQ-9 was a useful depression screener in this study population given the potential overlap of depressive symptoms with dialysis-related symptoms or if the PHQ-9 should be adjusted for such overlap before application. We found that the standard PHQ-9 was acceptable to use as a depression screener. More generally, in practice, clinicians should proceed with some caution when interpreting a test, questionnaire or other scale of interest in lieu of other comorbidities in patient care management. Full psychometric analyses using our advanced statistical methods can help determine if such overlap is problematic. Careful consideration of such overlap as well as clinical judgment is recommended when interpreting patient-reported outcomes and making care management decisions.

## Supplementary Material

Supplemental Material

Supplemental Material

## Figures and Tables

**Table:** B. hdPHQ-9

hdPHQ-9		MINI Diagnosis	
		+	−
Screening	+	17	25
	−	4	127

Sensitivity: 0.81 (0.58, 0.95); Specificity: 0.84 (0.77, 0.89); Pos Pred Value: 0.40 (0.26, 0.57); Neg Pred Value: 0.97 (0.92, 0.99).

## Data Availability

The data that support the findings of this study are not publicly available due to their containing information that could compromise the privacy of research participants but are available from DG upon reasonable request and IRB approval.

## Appendices

### Quality of life questionnaire

This survey asks for your views about your health. This information will help keep track of how you feel and how well you are able to do your usual activities.

This survey includes a variety of questions about your health and your life. We are interested in how you feel about each of these issues.

1. In general, would you say your health is:

**Table:**

Excellent	Very Good	Good	Fair	Poor
1	2	3	4	5
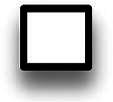

The following items are about activities you might do during a typical day. Does your health now limit you in these activities? If so, how much?

**Table:**

	Yes, limited a lot	Yes, limited a little	No, not limited at all
2. Moderate activities, such as moving a table, pushing a vacuum cleaner, bowling, or playing golf.	1	2	3
3. Climbing several flights of stairs.	1	2	3

During the past 4 weeks, have you had any of the following problems with your work or other regular daily activities as a result of your physical health?

**Table:**

	Yes	No
4. Accomplished less than you would like.	1	0
5. Were limited in the kind of work or other activities.	1	0

During the past 4 weeks, have you had any of the following problems with your work or other regular daily activities as a result of any emotional problems (such as feeling depressed or anxious)?

**Table:**

	Yes	No
6. Accomplished less than you would like.	1	0
7. Didn’t do work or other activities as carefully as usual.	1	0

8. During the past 4 weeks, how much did pain interfere with your normal work (including both work outside the home and housework)?

**Table:**

Not at all	A little bit	Moderately	Quite a bit	Extremely
1	2	3	4	5

These questions are about how you feel and how things have been with you during the past 4 weeks. For each question, please give the one answer that comes closest to the way you have been feeling.

How much of the time during the past 4 weeks…………

**Table:**

	All of the time	Most of the time	A good bit of the time	Some of the time	A little of the time	None of the time
9. Have you felt calm and peaceful?	1	2	3	4	5	6
10. Did you have a lot of energy?	1	2	3	4	5	6
11. Have you felt downhearted and blue?	1	2	3	4	5	6

12. During the past 4 weeks, how much of the time has your physical health or emotional problems interfered with your social activities (like visiting with friends, relatives, etc.)?

**Table:**

All of the time	Most of the time	Some of the time	A little of the time	None of the time
1	2	3	4	5

How true or false is each of the following statements for you?

**Table:**

	Definitely true	Mostly true	Don’t know	Mostly false	Definitely false
13. My kidney disease interferes too much with my life.	1	2	3	4	5
14. Too much of my time is spent dealing with my kidney disease.	1	2	3	4	5
15. I feel frustrated dealing with my kidney disease.	1	2	3	4	5
16. I feel like a burden on my family.	1	2	3	4	5

During the past 4 weeks, to what extent were you bothered by each of the following?

**Table:**

	Not at all bothered	Somewhat bothered	Moderately bothered	Very much bothered	Extremely bothered
17. Soreness in your muscles?	1	2	3	4	5
18. Chest pain?	1	2	3	4	5
19. Cramps?	1	2	3	4	5
20. Itchy skin?	1	2	3	4	5
21. Dry skin?	1	2	3	4	5
22. Shortness of breath?	1	2	3	4	5
23. Fainting spells	1	2	3	4	5
24. Dizziness?	1	2	3	4	5
25. Lack of appetite?	1	2	3	4	5
26. Washed out or drained?	1	2	3	4	5
27. Nausea or upset stomach?	1	2	3	4	5
28. Numbness in hands or feet?	1	2	3	4	5
29. Constipation?	1	2	3	4	5
30. Vomiting?	1	2	3	4	5
31. Diarrhea?	1	2	3	4	5
32. Bone or joint pain?	1	2	3	4	5
33. Problems with your access site?	1	2	3	4	5
34. Cough?	1	2	3	4	5
35. Dry mouth?	1	2	3	4	5
36. Headache?	1	2	3	4	5
37. Restless legs?	1	2	3	4	5
38. Stomach pain?	1	2	3	4	5
39. Back pain?	1	2	3	4	5
40. Feeling your heart pound or race?	1	2	3	4	5
41. Pain or problems during sexual intercourse?	1	2	3	4	5
42. Feeling nervous, anxious, or on edge?	1	2	3	4	5
43. Not being able to stop or control worrying?	1	2	3	4	5
44. Worrying too much about different things?	1	2	3	4	5
45. Trouble relaxing?	1	2	3	4	5
46. Being so restless that it is hard to sit still?	1	2	3	4	5
47. Becoming easily annoyed or irritable?	1	2	3	4	5
48. Feeling afraid as if something awful might happen?	1	2	3	4	5

**Effects of kidney disease on your daily life**

Some people are bothered by the effects of kidney disease on their daily life, while others are not. How much does kidney disease bother you in each of the following areas?

**Table:**

	Not at all bothered	Somewhat bothered	Moderately bothered	Very much bothered	Extremely bothered
49. Fluid restriction?	1	2	3	4	5
50.. Dietary restriction?	1	2	3	4	5
51. Your ability to work around the house?	1	2	3	4	5
52.. Your ability to travel?	1	2	3	4	5
53.. Being dependent on doctors and other medical staff?	1	2	3	4	5
54. Stress or worries caused by kidney disease?	1	2	3	4	5
55. Your sex life?	1	2	3	4	5
56. Your personal appearance?	1	2	3	4	5
