# Supplementary material for: Using latent variables to improve the management of depression among hemodialysis patients
Source: Ren Fail. 2024 Aug 1;46(2):2350767. doi: 10.1080/0886022X.2024.2350767 (PMC11299459; doi:10.1080/0886022X.2024.2350767)
Supplement: Supplemental Material [file IRNF_A_2350767_SM9882.docx]

**Supplementary Table Comparing Anxiety and Depression Screening in Study Participants with Baseline Data Collected Pre vs. Post-COVID Pandemic (Cutoff Date March 1, 2020).**

|  | *Pre-Covid (N = 915)* | *Post-Covid (N = 173)* | *p-value* |
| --- | --- | --- | --- |
| *Severity of Anxiety* | *10.44 ± 5.16* | *11.22 ± 5.42* | *0.035* |
| *Depression Screening* | *4.24 ± 4.52* | *6.18 ± 5.07* | *0.013* |

Mean ± Standard Deviation reported in the table as well as a p-value via two-tailed independent sample t-test. The PHQ-9 Total Score is our depression screener. We collected anxiety scores for the subjects using the items of the General Anxiety Disorder-7 (GAD-7) which measures the severity of anxiety [2]. However, since our study team asked these questions at the same time as the KDQOL and in order to ease the burden on study participants we used a similar 5 point Likert scale as the KDQOL instead of the 4 point Likert scale established in the GAD-7. Thus, we cannot adequately conclude what our total scores indicate in terms of severity of anxiety.
