# Supplementary material for: Using latent variables to improve the management of depression among hemodialysis patients
Source: Ren Fail. 2024 Aug 1;46(2):2350767. doi: 10.1080/0886022X.2024.2350767 (PMC11299459; doi:10.1080/0886022X.2024.2350767)

**Fig. 1 Model for Analysis of Overlapping Depressive Symptoms and Dialysis-Related Symptoms**


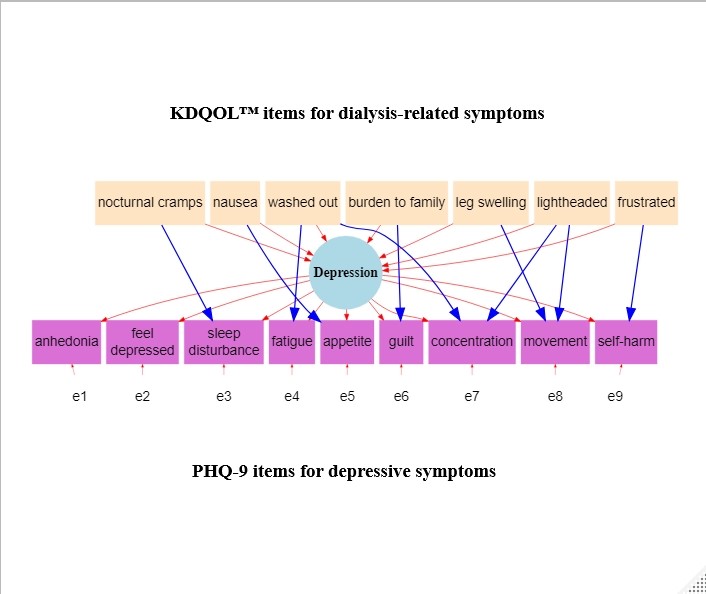


There are pairwise correlations among all the KDQOL items (nocturnal cramps, nausea, washed out, burden to family, leg swelling, lightheaded and frustrated) in the specified model that we omit from the figure due to visual complexity.

**Supplementary Fig. 1. Density Histogram of the PHQ-9 Total Score in our Study Sample of Dialysis Patients (N = 1085).**


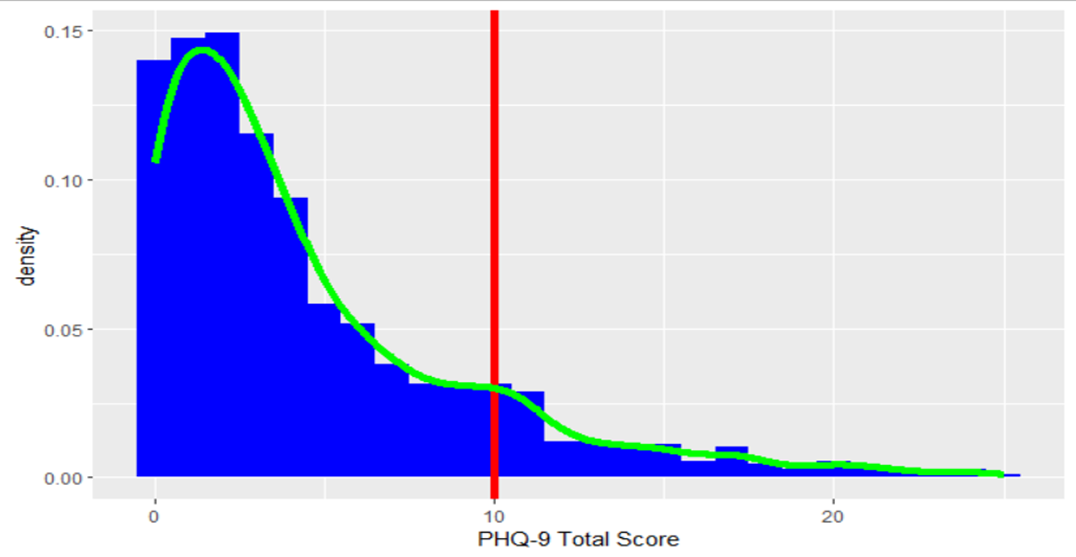

Supplement: Supplemental Material [file IRNF_A_2350767_SM9881.docx]
